# Ultrathin and Flexible CNTs/MXene/Cellulose Nanofibrils Composite Paper for Electromagnetic Interference Shielding

**DOI:** 10.1007/s40820-019-0304-y

**Published:** 2019-09-07

**Authors:** Wentao Cao, Chang Ma, Shuo Tan, Mingguo Ma, Pengbo Wan, Feng Chen

**Affiliations:** 1 Department of Orthopedics, Shanghai Tenth People’s Hospital, Tongji University School of Medicine, Shanghai, 200072 People’s Republic of China; 2 Engineering Research Center of Forestry Biomass Materials and Bioenergy, Beijing Key Laboratory of Lignocellulosic Chemistry, College of Materials Science and Technology, Beijing Forestry University, Beijing, 100083 People’s Republic of China; 3 Center of Advanced Elastomer Materials, State Key Laboratory of Organic-Inorganic Composites, Beijing University of Chemical Technology, Beijing, 100029 People’s Republic of China

**Keywords:** MXene, Carbon nanotubes, Cellulose nanofibrils, Mechanical property, Electromagnetic interference shielding

## Abstract

As the rapid development of portable and wearable devices, different electromagnetic interference (EMI) shielding materials with high efficiency have been desired to eliminate the resulting radiation pollution. However, limited EMI shielding materials are successfully used in practical applications, due to the heavy thickness and absence of sufficient strength or flexibility. Herein, an ultrathin and flexible carbon nanotubes/MXene/cellulose nanofibrils composite paper with gradient and sandwich structure is constructed for EMI shielding application via a facile alternating vacuum-assisted filtration process. The composite paper exhibits outstanding mechanical properties with a tensile strength of 97.9 ± 5.0 MPa and a fracture strain of 4.6 ± 0.2%. Particularly, the paper shows a high electrical conductivity of 2506.6 S m^−1^ and EMI shielding effectiveness (EMI SE) of 38.4 dB due to the sandwich structure in improving EMI SE, and the gradient structure on regulating the contributions from reflection and absorption. This strategy is of great significance in fabricating ultrathin and flexible composite paper for highly efficient EMI shielding performance and in broadening the practical applications of MXene-based composite materials.

**Electronic supplementary material:**

The online version of this article (10.1007/s40820-019-0304-y) contains supplementary material, which is available to authorized users.

## Highlights

- An ultrathin and flexible carbon nanotubes/MXene/cellulose nanofibrils composite paper with gradient and sandwich structure was successfully fabricated via a facile alternating vacuum-assisted filtration process.
- The composite paper exhibits excellent mechanical property and electromagnetic interference shielding performance.

**Electronic supplementary material:**

The online version of this article (10.1007/s40820-019-0304-y) contains supplementary material, which is available to authorized users.

## Graphical abstract

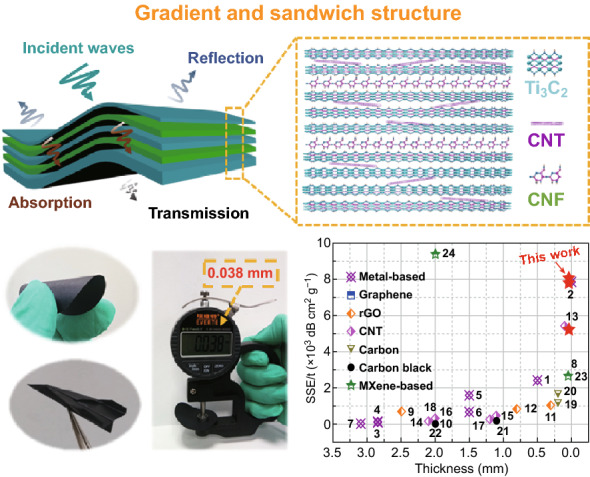

## Introduction

With the prosperity and development of electronics technology, functional electromagnetic materials and devices have been extensively used in diverse fields, ranging from household electrical appliances to military weapons [1, 2]. High-efficiency electromagnetic interference (EMI) shielding materials are of great significance for the alleviation or elimination of electromagnetic radiation pollution that has potential detrimental effects on human health and the normal work of electronic equipment [3–7]. As typical carbon-based materials, one-dimensional (1D) carbon nanotubes (CNTs) and two-dimensional (2D) graphene sheets have drawn a wide of attention for EMI shielding owing to their great superiorities in lightweight and flexibility, in contrast to the old-fashioned heavy and vulnerable metal (e.g., Cu, Ni, and Ag) materials [8–11]. For example, Song et al. [4] fabricated a lightweight and conductive CNTs-multilayered graphene edge plane core–shell hybrid foam for EMI shielding using a chemical vapor deposition method. The resultant hybrid foam exhibited high EMI shielding effectiveness with more than 38.4 dB in X-band. Although many exciting advances have been made, it is still a considerable challenge for carbon-based materials to achieve a self-supported conductive network with an efficient EMI shielding performance at small thickness [12–14].

A novel family of 2D early transition metal carbides and/or nitrides, MXenes (*M*_*n*+1_*X*_*n*_*T*_*x*_, where *M* represents an early transition metal, *X* represents C and/or N, *n* = 1, 2, or 3, and *T* is a terminating group, such as O, OH, and/or F), were firstly discovered in 2011 by the selective etching and delamination of their layered MAX phase [15–19]. With large specific surface area and high electrical conductivity [20–25], Ti_3_C_2_T_*x*_ MXene has recently been extensively reported as a remarkable shielding material [26–39]. For example, Gogotsi and his co-workers firstly reported a metallically conductive Ti_3_C_2_T_*x*_-based film with excellent conductivity (4665 S cm^−1^) and superior EMI shielding effectiveness (> 92 dB, 45 µm) [3]. Sun et al. [40] subsequently fabricated a highly conductive MXene/polystyrene nanocomposite with the electrostatic assembly method. The resultant nanocomposite reached a maximum EMI SE of 62 dB at a low MXene loading of 1.90 vol%. Li et al. [41] have newly reported a reduced graphene oxide/Ti_3_C_2_T_*x*_ hybrids foam with hollow core–shell architecture, which exhibits an excellent EM absorption performance. Although some impressive progress has been made, the fabrication of EMI materials with ultrathin thickness, high flexibility, and excellent EMI shielding performance is still an enormous challenge.

Cellulose nanofibrils (CNFs), which can be isolated by (2,2,6,6-tetramethylpiperidin-1-yl) oxidanyl (TEMPO) oxidation and mechanical processing from wood and plants, are composed of numerous aligned *β*-*D*-(1 → 4)glucopyranose polysaccharide chains with abundant strongly intermolecular hydrogen bonds [42–45]. With excellent mechanical toughness, integrating high stiffness about 140 GPa and a lightweight character [46–48], CNFs have been considered as a promising emerging class of high-performance nature-derived nanomaterials [49–52]. Moreover, CNFs with a typical 1D nanofiber structure will generate less insulating contacts between conductive nanosheets, such as reduced graphene oxide nanosheets [53], boron nitride nanosheets [54], and MXene nanosheets [55]. Particularly, CNFs are globally abundant, renewable, and environmental friendly since they are extracted from plants (e.g., wood, cotton, garlic husk). Yang et al. [53] fabricated reduced graphene/cellulose nanofiber composite films with great thermal conductivity and EMI shielding performance by vacuum-assisted filtration and hydroiodic acid reduction process. Zhang et al. [56] reported the preparation of cellulose nanofibers/multiwalled carbon nanotube composite film for EMI shielding via vacuum filtration and hot-pressing method. Recently, our group has reported a highly flexible MXene/cellulose nanofiber composite paper via a vacuum-assisted filtration self-assembly process [55]. However, owing to the insulating character of cellulose, the composite paper prepared by direct mixing of MXene and cellulose nanofibrils has showed a slight reduction in the conductivity and EMI shielding performance [53, 57, 58]. Thus, it is highly desired to develop a facile synthetic strategy, which can simultaneously improve the mechanical property, electrical conductivity, and EMI shielding performance of MXene-based composite materials [59, 60].

In this work, we report an ultrathin and flexible CNTs/Ti_3_C_2_ MXene/CNFs composite paper with gradient and sandwich structure (CMC GS) for next generation of EMI shielding application by a facile alternating vacuum-assisted filtration method. The CMC GS composite paper with excellent mechanical properties shows a combination of high tensile strength and toughness. Furthermore, the CMC composite paper with gradient and sandwich structure shows a high electrical conductivity of 2506.6 S m^−1^ and an enhanced EMI SE of 38.4 dB. The gradient structure of the composite paper plays a crucial role in regulating its contributions from reflection and absorption, rather than its total EMI shielding effectiveness. In contrast to gradient structure, the sandwich structure possesses more favorable advantages in the improvement in EMI SE of composite paper. Thus, the novel structural design in the fabrication of ultrathin and flexible composite paper contributes to realize outstanding EMI shielding performance and will broaden the practical applications of MXene composite materials.

## Experimental Section

### Materials

Lithium fluoride (LiF, ≥ 99%), cetyltrimethylammonium bromide (CTAB, > 99%), sodium bromide (NaBr), and sodium hypochlorite (NaClO) were purchased from Aladdin Industrial Corporation. Hydrochloric acid (HCl, 37 wt%) and sodium hydroxide (NaOH) were obtained from Sinopharm Chemical Reagent Co., Ltd. CNTs (diameter 5–15 nm) were obtained from Shenzhen Nanotech Co., Ltd., of China. Ti_3_AlC_2_ powders were purchased from Jilin 11 technology Co., Ltd. The bleached softwood pulp was purchased from Donghua Pulp Factory, China. All reagents and chemicals were used as received without further purification.

### Synthesis of Ti_3_C_2_ MXene Nanosheets

Ti_3_C_2_ nanosheets were synthesized according to a wet chemical etching method as previously reported by Gogotsi [3, 61]. Typically, 1 g of LiF was dissolved in 20 mL HCl (9 M) in a Teflon vessel and stirred for about 30 min at room temperature (RT). Then, 1 g of Ti_3_AlC_2_ powders was slowly added into the etchant solution and the mixture was allowed to proceed at 35 °C for 24 h under stirring. The resultant slurry was washed repeatedly using deionized (DI) water and centrifuged at 3500 rpm for 5 min until its pH reaches about 6. The black swelled clay-like sediment was re-dispersed and further exfoliated in deionized (DI) water under vigorously shaking for about 10 min. Finally, the uniform delaminated Ti_3_C_2_ MXene nanosheets suspension with a concentration of 0.5 mg mL^−1^ was obtained after centrifuging for 1 h at 3500 rpm.

### Preparation of Single-Layered Ti_3_C_2_–CNTs Composite Paper

CNTs aqueous dispersion with a concentration of 0.1 mg mL^−1^ was produced by ultrasonication for 20 min in the presence of cationic surfactant CTAB. CNTs dispersion was added dropwise to Ti_3_C_2_ nanosheets suspension followed by further ultrasonication for 10 min to guarantee the complete contacts. The mixed suspension was filtered using a cellulose filter (0.22 µm in pore size) and dried at 60 °C, achieving the single-layered Ti_3_C_2_/CNTs composite paper. The weight ratios of CNTs–Ti_3_C_2_ chosen were 1:5, 1:10, and 1:15, and the resulting single-layered composite papers were denoted as CM-5, CM-10, and CM-15, respectively. In these cases, the mass of the CNTs was set as 1 mg.

### Fabrication of TEMPO-Mediated Oxidized Cellulose Nanofibrils (CNFs)

CNFs were prepared using a known method [43, 62]. First, TEMPO (100 mg) and NaBr (659 mg) were added into a softwood pulp suspension (100 mL, 1 wt%) in a glass beaker. Then, 38 mL of 12% NaClO was added into the above mixture slowly at RT to initiate TEMPO-mediated oxidation. During the above oxidation process, the pH value of the mixture was maintained at 10.5 with 0.5 M NaOH until no pH variation was observed. The TEMPO-oxidized cellulose was purified by washing several times with DI water. The product slurry was re-dispersed in DI water and further treated with a blender machine. The nanofibers were separated from unexfoliated cellulose fibers with high-speed centrifugation for 30 min. Finally, the collected supernatant was further treated with a high pressure homogenizer to obtain the uniform cellulose nanofibrils dispersion. The concentration of the CNFs was regulated as 0.5 mg mL^−1^.

### Preparation of CNTs/Ti_3_C_2_ MXene/CNFs Composite Paper with Gradient and Sandwich Structure (CMC GS)

The GMT GS composite paper was prepared using an alternating vacuum-assisted filtration method. For instance, the CM-5 was firstly filtered on the filter membrane to form a thin continuous layer. Then, a CNFs (4 mg) layer was deposited on the top of CM-5 in the same way. After that, CM-10, CNFs (4 mg), and CM-15 were successively added to be deposited on their former layer. The composite paper was then dried in vacuum oven at 60 °C and peeled off from the filter membrane, yielding free-standing CMC GS composite paper. Other composite papers with gradient or/and sandwich structure can be prepared through a similar process. To make the comparison, a randomly mixed CNTs (3 mg)**/**Ti_3_C_2_ MXene (30 mg)/CNFs (8 mg) composite paper, which was labeled as CMC mixture, was also constructed by a similar vacuum-assisted filtration of mixed CNTs**/**Ti_3_C_2_ MXene/CNFs dispersion.

### Characterization and Measurements

Field emission scanning electron microscopy (SEM, S4800, Hitachi, Japan) and transmission electron microscopy (TEM, Hitachi H-800, Japan) were used to characterize morphologies and microstructures of the samples. The phase compositions of the samples were analyzed by X-ray diffractometer (Rigaku D/max 2550 V, Cu Kα radiation, *λ* = 1.54178 Å). Fourier-transform infrared (FTIR) spectroscopy was measured using a FTIR spectrometer (FTIR-7600, Lambda Scientific, Australia). The surface chemistries of the samples were characterized by X-ray photoelectron spectroscopy (XPS, ESCALAB 250Xi, Thermo Scientific). Nitrogen sorption measurements were obtained at − 196 °C on a Quadrasorb instrument (Quantachrome, USA) to measure the specific surface area and pore size distribution. The mechanical tests were performed at RT by a universal testing machine (Zwick Z005) equipped with a 100 N load cell. Each sample was cut into strips (10 × 30 mm^2^) using a knife blade, and the loading rate was set as 0.2 mm min^−1^. The electrical conductivity measurements were conducted at RT using a physical property measurement system (Quantum Design) with the standard four-probe method. All samples were cut into strips (2.5 × 2.0 mm^2^) for measurements. Four-pin probe was tightly contacted with the samples, and sheet resistance was recorded. The electrical conductivity of samples was calculated as Eq. 1:

1 $$\sigma = \frac{1}{s} \cdot \frac{1}{{{\raise0.7ex\hbox{$R$} \!\mathord{\left/ {\vphantom {R L}}\right.\kern-0pt} \!\lower0.7ex\hbox{$L$}}}} = \frac{L}{R \cdot w \cdot t}$$

where *σ* is the electrical conductivity (*S* cm^−1^), *R* is the sheet resistance (Ω sq^−1^), *L* and *S* are the length (cm) and cross-sectional area (cm^2^) of the measured samples, and *w* and *t* are the width (cm) and thickness (cm) of the samples.

Agilent PNA-N5244A vector network analyzer was employed to measure the electromagnetic interference shielding effectiveness (EMI SE) of samples in the frequency range of 8.2–12.4 GHz on the basis of a waveguide method. The samples were cut into the rectangular shape with a dimension of 22.9 × 10.2 mm^2^ for measurements. The reflection (*R*), transmission (*T*), and absorption (*A*) coefficients were obtained by calculating the scattering parameters (*S*_11_ and *S*_21_). The total electromagnetic interference shielding values (SE_*T*_) can be obtained by Eqs. 2–7:

2 $${\text{SE}}_{T} = {\text{SE}}_{A} + {\text{SE}}_{R} + {\text{SE}}_{M}$$

3 $$R + A + T = 1$$

4 $$R = \left| {S_{11} } \right|^{2} = \left| {S_{22} } \right|^{2}$$

5 $$T = \left| {S_{12} } \right|^{2} = \left| {S_{21} } \right|^{2}$$

6 $${\text{SE}}_{R} = 10\log \left( {\frac{1}{1 - R}} \right) = 10{ \log }\left( {\frac{1}{{1 - \left| {S_{11} } \right|^{2} }}} \right)$$

7 $${\text{SE}}_{A} = 10\log \left( {\frac{1 - R}{T}} \right) = 10{ \log }\left( {\frac{{1 - \left| {S_{11} } \right|^{2} }}{{\left| {S_{21} } \right|^{2} }}} \right)$$

where SE_*R*_ is the reflection value, SE_*A*_ is the absorption value, and SE_*M*_ is the multiple internal reflection value. The SE_*M*_ can be negligible at the time of SE_*T*_ ≥ 15 dB [10, 63, 64]. To compare the effectiveness of shielding materials equitably, density and thickness of the materials were also taken into account. The related equations were described as:

8 $${\text{SSE}} = \frac{{{{\rm EMI }}\,{{\rm SE}}}}{{\rm density}} = {\text{dB}}\, {\text{cm}}^{3} \, {\text{g}}^{ - 1}$$

9 $${\text{SSE}}/t = \frac{{\rm SSE}}{{\rm thickness}} = {\text{dB}}\, {\text{cm}}^{2} \,{\text{g}}^{ - 1}$$

The EMI shielding efficiency (%) can be obtained as Eq. 10:

10 $${\text{Shielding }}\,{\text{effiency}}\, \left( {\text{\% }} \right) = 100 - \left( {\frac{1}{{10^{{\frac{\text{SE}}{10}}} }}} \right) \times 100$$

## Results and Discussion

**Fig. 1:**
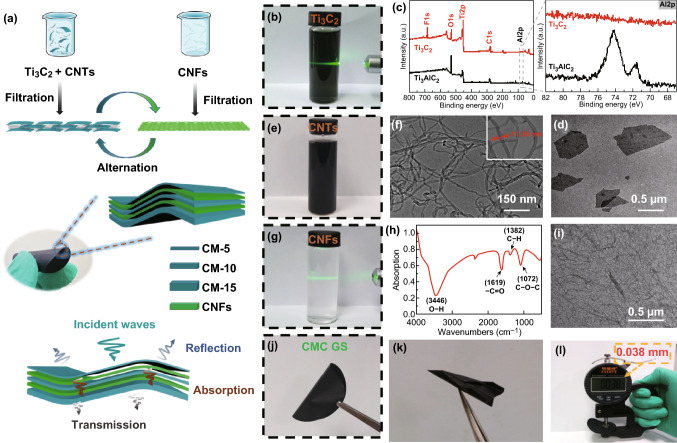
**a** Schematic illustrating the fabrication of CMC GS composite paper. **b** Digital image, **c** XPS spectra, and **d** TEM image of Ti_3_C_2_ MXene. **e** Digital image and **f** TEM image of CNTs. **g** Digital image, **h** FTIR spectra, and **i** TEM image of CNFs. **j**–**l** Digital images of the as-prepared CMC GS composite paper

Figure 1a schematically illustrates the preparation process of CMC GS composite paper. First, a thin continuous layer of CNTs/Ti_3_C_2_ MXene (CM) can be obtained by a vacuum-assisted filtration from a dispersed CM aqueous solution containing 1D CNTs and 2D Ti_3_C_2_ nanosheets. Subsequently, a CNFs layer deposited on the top of CM layer is obtained in the same way. Afterward, this filtration process is repeated several times alternately with various Ti_3_C_2_ contents. By this strategy, CMC GS composite paper can be facilely obtained without using any expensive equipment or toxic organic solvents, which may have an enormous potential for large-scale manufacturing. Particularly, the introduction of gradient and sandwich structure in the composite paper is in favor of the simultaneous enhancement of mechanical properties and EMI shielding performance. For purpose of comparison, a free-standing randomly mixed CNTs/Ti_3_C_2_ MXene/CNFs (CMC mixture) composite paper without gradient and sandwich structure has also been produced by the direct vacuum-assisted filtration of CNTs/Ti_3_C_2_ MXene/CNFs mixed dispersion.

2D Ti_3_C_2_ MXene nanosheets were prepared by selectively etching Ti_3_AlC_2_ precursor (MAX phase) with HCl/LiF and further delaminating under vigorously manual shaking (Fig. S1). SEM images show that Ti_3_AlC_2_ MAX phase is a closely stacked ternary compound (Fig. S2a, b). Ti_3_AlC_2_ solid bulk changes to loosely stacked multilayered Ti_3_C_2_ MXene with a characteristic accordion-like structure (Fig. S2c, d). After further delaminating, Ti_3_C_2_ MXene nanosheets dispersion with a typical Tyndall effect was observed (Fig. 1b). As shown in X-ray diffraction (XRD) patterns, the representative (002) peak shifts from 9.6° to 7.0°, indicating the increased interlayer spacing from 9.20 to 12.6 Å (Fig. S3) [23, 65]. The chemical composition and surface terminations of Ti_3_AlC_2_ MAX phase and Ti_3_C_2_ nanosheets were determined by XPS (Fig. 1c). XPS results exhibit the absence of Al element and the existence of Ti–C (2p3) and Ti–O (2p3) doublets, which are in accordance with the previous reports and indicate the successful preparation of Ti_3_C_2_ nanosheets (Fig. S4) [66, 67]. The TEM image shows that delaminated Ti_3_C_2_ nanosheets with a diameter of ~ 500 nm were ultrathin and nearly transparent (Figs. 1d and S5). In addition, the dispersed CNTs aqueous solution with a concentration of 0.1 mg mL^−1^ has also been produced by an ultrasonic processing with the assistance of cationic surfactant CTAB (Fig. 1e) [68]. The CNTs with 5–15 nm in diameter and 15–30 µm in length (Fig. 1f) have been employed to enhance the connectivity between Ti_3_C_2_ nanosheets by forming a porous conductive network. When CNTs are added to Ti_3_C_2_ dispersion, Ti_3_C_2_ nanosheets are connected together to form numerous aggregations (Fig. S6). Figure 1g shows the homogeneously dispersed cellulose nanofibrils with Tyndall effect prepared from wood pulp with the TEMPO-mediated oxidation method. FTIR spectroscopy measurement has been performed on the CNFs, and the result is presented in Fig. 1h. The FTIR spectrum shows a broad and strong absorption band at 3446 cm^−1^, which is ascribed to the stretching vibration of O–H, while the strong absorption bands at 1619 cm^−1^ are assigned to C=O, proving the successful TEMPO-mediated oxidation process. TEM measurement has been taken to further observe the micromorphology of CNFs (Fig. 1i). TEM image shows that the diameters of CNFs are 10–50 nm and lengths are 200–500 nm. After alternant vacuum-assisted filtration of CM and CNFs, a self-supported and flexible CMC GS composite paper has been obtained. As shown in Fig. 1j, k, the composite paper can be folded into an intricate “airplane” shape without any crack or fracture, indicating excellent flexibility. Moreover, Fig. 1l shows that the thickness of the CMC GS composite paper is only 0.038 mm, which presents a great potential in miniaturized and lightweight electronic devices.

**Fig. 2:**
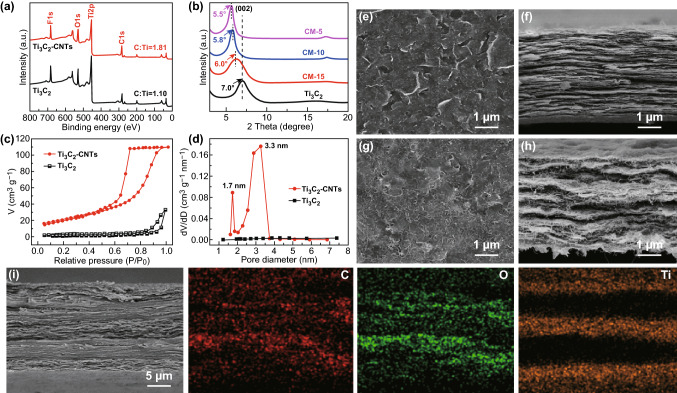
**a** XPS survey spectrum of the pure Ti_3_C_2_ paper and Ti_3_C_2_–CNTs composite paper. **b** XRD patterns of the Ti_3_C_2_–CNTs composite paper with different Ti_3_C_2_ contents. **c**, **d** N_2_ sorption/desorption isotherms and pore size distribution curves of pure Ti_3_C_2_ paper and Ti_3_C_2_–CNTs composite paper. **e** Top-view and **f** cross-sectional SEM images of pure Ti_3_C_2_ paper. **g** Top-view and **h** cross-sectional SEM images of Ti_3_C_2_–CNTs composite paper. **i** Cross-sectional SEM and EDS mapping images of CMC GS composite paper

The chemical compositions of the single-layered Ti_3_C_2_ and Ti_3_C_2_–CNTs composites have been analyzed by XPS spectra. As shown in Fig. 2a, both pure Ti_3_C_2_ paper and Ti_3_C_2_–CNTs composite paper have abundant F– and O– groups, which are attributed to the fluorine and oxygen terminations on Ti_3_C_2_ nanosheets. However, the Ti_3_C_2_–CNTs composite paper has a larger C/Ti atomic ratio (1.81) than that of the pure Ti_3_C_2_ paper (1.10), owing to the addition of CNTs. Figure 2b shows the XRD patterns of Ti_3_C_2_ and Ti_3_C_2_–CNTs composite papers with different Ti_3_C_2_ contents obtained by the vacuum-assisted filtration method. The retained (002) peaks of CM-5, CM-10, and CM-15 with various Ti_3_C_2_ contents (CM-5: Ti_3_C_2_–CNTs weight ratio is 5:1; CM-10: Ti_3_C_2_–CNTs weight ratio is 10:1, and CM-15: Ti_3_C_2_–CNTs weight ratio is 15:1) indicated the well-preserved laminated structure of 2D Ti_3_C_2_ nanosheets. Moreover, with decreasing content of Ti_3_C_2_, the (002) peak shifts from 2*θ* = 7.0° to 5.5°, signifying that the *d*-spacing of Ti_3_C_2_ nanosheets clearly increases from approximately 12.6 Å of pure Ti_3_C_2_ paper to 16.0 Å of CM-5 composite paper. The increased *d*-spacing is attributed to the successful intercalation of the CNTs into the interlayer spaces between Ti_3_C_2_ nanosheets.

To further explore the role of CNTs acted in Ti_3_C_2_–CNTs composite paper, N_2_ sorption/desorption measurements have been employed to characterize their microstructure and exposed specific surface area. Figure 2c exhibits the N_2_ sorption isotherms of pure Ti_3_C_2_ paper and Ti_3_C_2_–CNTs composite paper. Unlike the pure Ti_3_C_2_ paper, Ti_3_C_2_–CNTs composite paper shows a typical type-V behavior with an obvious hysteresis loop of type H_2_, which is resulted from the micropores generated through the introduction of CNTs between Ti_3_C_2_ nanosheets. The specific surface area calculated based on Brunauer–Emmett–Teller (BET) method shows that the pure Ti_3_C_2_ paper has a small BET surface area of 7.2 m^2^ g^−1^. On the contrary, Ti_3_C_2_–CNTs composite paper possesses a larger BET surface area of 77.2 m^2^ g^−1^. The increased BET surface area indicates that the introduction of CNTs effectively prevents the restacking effect and maximizes the accessibility of 2D Ti_3_C_2_ nanosheets. As shown in Figs. 2d and S7, besides the uniform pore size distribution of CNTs about 1.7 nm, the Ti_3_C_2_–CNTs composite paper also has a pore size distribution within the scope of 2.1–3.7 nm. In contrast, the pure Ti_3_C_2_ paper lacks these micropores and mesopores in this range. To understand the role of CNTs in Ti_3_C_2_–CNTs composite more intuitively, SEM equipped with energy-dispersive spectroscope elemental (EDS) mappings measurements is performed to characterize their morphologies and elements distribution. Figure 2e, f exhibits the top-view and cross-sectional SEM images of the pure Ti_3_C_2_ paper. Without the incorporation of CNTs, the pure Ti_3_C_2_ paper displayed a compactly stacked lamellar structure without visible pore. In contrast, after the introduction of CNTs as spacers, 2D Ti_3_C_2_ nanosheets achieve effective separation and avoided the restack, yet still connected sufficiently to form a continuous conductive network (Fig. 2g). Moreover, the Ti_3_C_2_ nanosheets have a uniform distribution in Ti_3_C_2_–CNTs composite paper, which are observed in EDS mapping results (Fig. S8). Besides, it is worth mentioning that the Ti_3_C_2_–CNTs composite paper with an undulating layered structure has numerous slit-shaped micropores (Figs. 2h and S9), which can be ascribed to the elimination of Ti_3_C_2_ nanosheets restacking by CNTs. The undulating layered structure and slit-shaped micropores of composite layer are of great significance for increasing the reflection and absorption of electromagnetic waves and further enhancing its EMI shielding performance. Single-layered CM and CNFs are used to assemble into a CMC GS composite paper in the alternant vacuum-assisted filtration process. Figure 2i shows the cross-sectional SEM and EDS mapping images, which indicate the CNTs/Ti_3_C_2_ MXene/CNFs composite paper with gradient and sandwich structure has been successfully obtained. Meanwhile, a free-standing CMC mixture composite paper with a homogeneous distribution of Ti_3_C_2_ nanosheets has been prepared as a control by the directing vacuum-assisted filtration process, which displays a uniform composite structure (Fig. S10).

**Fig. 3:**
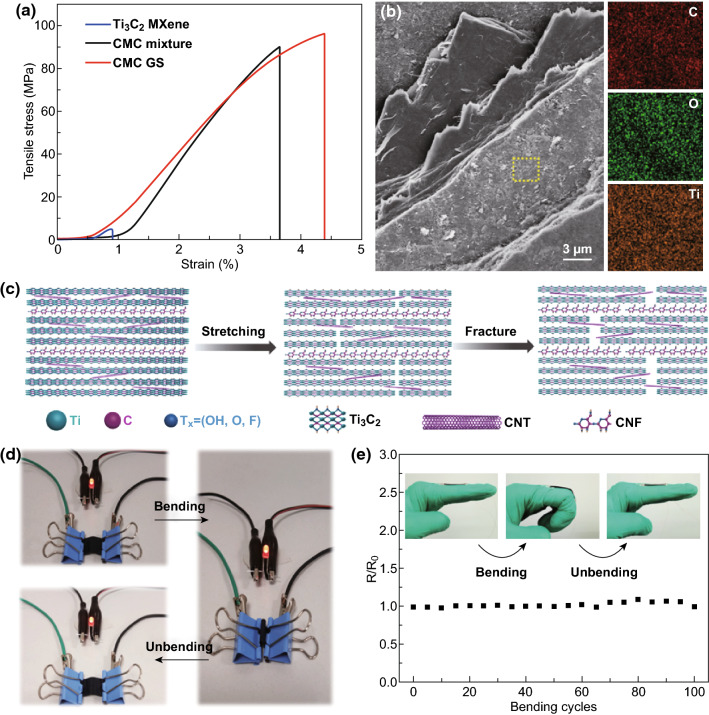
**a** Tensile stress–strain curves of the pure Ti_3_C_2_ MXene, CMC mixture, and CMC GS composite paper. **b** SEM image and the corresponding EDS map of the fracture surface of CMC GS composite paper. **c** Schematic illustration of the crack propagation mode of CMC GS composite paper. **d**, **e** Electrical resistance variation of CMC GS composite paper with bending test

Mechanical property is of great significance for EMI shielding materials, especially in the field of wearable or portable electronic devices, which need sufficient flexibility to endure mechanical deformation. It can be seen that CMC GS composite paper is stable after several foldings and being pressed with a weight of 200 g (Fig. S11). The typical tensile stress–strain curves of the pure Ti_3_C_2_ MXene, CMC mixture, and CMC GS composite paper are revealed in Fig. 3a, and the detailed data of mechanical performance are provided in Table S1. The tensile strength of the pure Ti_3_C_2_ MXene paper is only 4.9 ± 1.0 MPa, with a fracture strain of 0.9 ± 0.1% (Fig. S12). The pure Ti_3_C_2_ MXene paper with poor mechanical properties is hard to meet the requirement for practical applications. In contrast, the randomly assembled CMC mixture composite paper exhibits an improved mechanical property with a tensile strength of 94.9 ± 7.4 MPa and a fracture strain of 3.6 ± 0.2%. The significant improvement in tensile stress and strain can be attributed to the successful incorporation of CNFs, which is often used as an ideal reinforcement. Particularly, the CMC GS composite paper, which is prepared by the alternant filtration of CNFs and CM with various Ti_3_C_2_ contents, is not seen any reduction in mechanical properties. For instance, the CMC GS composite paper shows a tensile strength of 97.9 ± 5.0 MPa, a fracture strain of 4.6 ± 0.2%, a toughness of 2.1 ± 0.2 MJ m^−3^, and a Young’s modulus of 2.6 ± 0.2 GPa. Moreover, the mechanical performances of the Ti_3_C_2_–CNTs (*W*_MXene_/*W*_CNTs_ = 5:1) composite paper and the pure CNFs paper have been also investigated (Fig. S13). Compared to Ti_3_C_2_–CNTs composite paper with poor mechanical property, the pure CNFs paper exhibits a great mechanical performance with a tensile strength of 95.7 ± 13.7 MPa and a fracture strain of 5.1 ± 1.7%. The striking contrast between fragile Ti_3_C_2_–CNTs composite paper and flexible pure CNFs paper further confirms the strengthening effect of CNFs in CMC GS composite paper.

To investigate the fracture mechanism of the CMC GS composite paper, the SEM image for the fracture surface is shown in Fig. 3b. The surface of CMC GS composite paper shows obvious hierarchical fracture, which can be ascribed to the uneven and hierarchical distribution of CNFs and CM. Moreover, the fracture surface of the CMC GS composite paper shows a long-range crack deflection and “pull-out” mode, indicating that an additional friction exists in contact surfaces owing to the inter-limitation between CNFs and CM. EDS mapping, which has been conducted on one of the convex surfaces within the fracture surface of CMC GS composite paper, shows an even distribution of C, O, and Ti elements, confirming that the “pull-out” mode exists in stretching process. As shown in Fig. 3c, a crack propagation mode of CMC GS composite paper is proposed to explain the mechanism. When the composite paper is subjected to tensile load, the adjacent Ti_3_C_2_ nanosheets can be inclined to slide over each other, and the hydrogen bonds between the CM layer and CNFs layer are slowly destroyed, which resulted in an initial crack. Subsequently, the long-chain CNFs molecules are stretched along the drawing direction to dissipate more energy during the further stretching process, until the composite paper realizes a completely fracture. During the fracture process, the gradient and sandwich structure of the composite paper contributed to an obvious hierarchical fracture. To explore the electrical resistance variation of CMC GS composite paper under bending, the composite paper is fixed on two insulating clips and then linked with a light-emitting diode (LED) lamp. As intuitively reflected by the images in Fig. 3d, the brightness of LED lamp is not found any observable change during 100 times bending (Movie S1). In addition, we have further investigated the potential applications of CMC GS composite paper in the field of wearable designs. The composite paper is attached to index finger by conductive silver paste to monitor resistance variation after several cycles of finger bending tests. As shown in Fig. 3e, the resistance exhibits no conspicuous increase even after 100 bending cycles. The great flexibility and stable conductivity of CMC GS composite paper indicate the significant potentials in practical application to wearable or portable electronic devices.

**Fig. 4:**
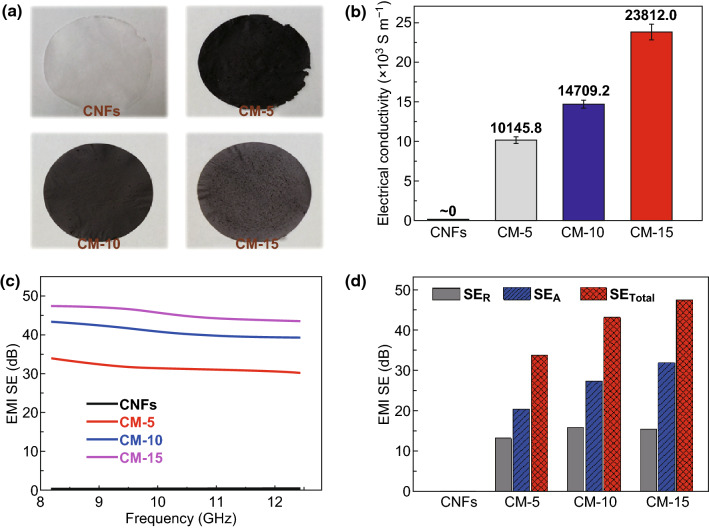
**a** Digital images of single-layered CNFs, CM-5, CM-10, and CM-15. **b** Electrical conductivity and **c** EMI SE of single-layered CNFs, CM-5, CM-10, and CM-15 in the X-band region. **d** Comparison of SE_Total_, SE_*A*_, and SE_*R*_ of single-layered CNFs, CM-5, CM-10, and CM-15 at the frequency of 8.2 GHz

The images of single-layered CNFs, CM-5, CM-10, and CM-15, which are used to be assembled into CMC GS composite paper, are shown in Fig. 4a. As we all know, the EMI shielding performance for the conductive materials is of great relevance to their electrical conductivity. It can be seen easily from Fig. 4b that the single-layered CNFs are an insulating material with no electrical conductivity. The single-layered CM-5 with a Ti_3_C_2_–CNTs weight ratio of 5:1 displays an electrical conductivity of 10,145.8 S m^−1^, which is about 10,000 times more than the requirement (1 S m^−1^) for EMI shielding materials in actual applications [53]. By increasing the Ti_3_C_2_–CNTs weight ratio to 15:1, the obtained single-layered CM-15 realizes an ultrahigh electrical conductivity of 23,812.0 S m^−1^. The excellent electrical conductivity of single-layered CM can be attributed to the continuous conductive network formed by 1D CNTs and 2D Ti_3_C_2_ nanosheets.

As expected, the superior conductivity endows the single-layered CM with excellent EMI shielding performance (Fig. 4c). The total EMI shielding performance of single-layered CM is improved with the increment in Ti_3_C_2_ content, following a parallel tendency to the variation of electrical conductivities. Moreover, all the single-layered CM samples show excellent EMI shielding effectiveness (SE) of > 30 dB over the whole X-band. In particular, comparing with a 15-mg-pure Ti_3_C_2_ paper with relative lower average EMI SE of 34 dB (Fig. S14), the single-layered CM-15 exhibits a superb EMI SE value of > 43 dB over the whole X-band with a maximum of 48 dB, which is mostly owing to the above-mentioned undulating layered structure and slit-shaped micropores of the single-layered CM. To theoretically clarify the EMI shielding mechanism of the single-layered CM, the total EMI shielding effectiveness (SE_Total_), microwave reflection (SE_*R*_), and microwave absorption (SE_*A*_) all over the X-band have been investigated, as shown in Fig. S15. With increasing the content of Ti_3_C_2_ in single-layered CM, SE_*A*_ exhibits an obvious ascending trend, whereas SE_*R*_ does not show any significant variation. The inferior microwave reflection and strong microwave absorption indicate an absorption dominant shielding mechanism. For example, the SE_Total_, SE_*A*_, and SE_*R*_ of the single-layered CM-15 at the frequency of 8.2 GHz are 47.6, 31.9, and 15.7 dB, respectively, which show that the contribution of reflection to total EMI SE (33%) is much lower than that from absorption (67%) (Fig. 4d).

**Fig. 5:**
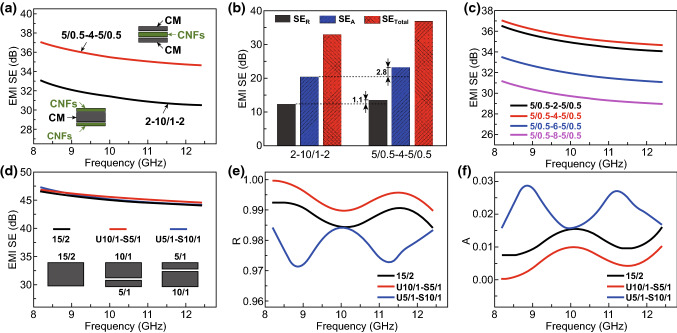
**a** EMI SE of composite paper with different sandwich structures in the X-band. **b** Comparison of SE_Total_, SE_*A*_, and SE_*R*_ of composite paper with different sandwich structures. **c** The EMI SE of CM–CNFs–CM composite paper with various CNFs contents in the X-band. The **d** EMI SE, **e** *R*, and **f** *A* of evenly distributed CM composite paper and two-layered CM composite paper with different gradient structures in the X-band

To quantitatively analyze the influence of sandwich structure on EMI SE enhancement of composite paper, a CM–CNFs–CM composite paper with a symmetric layered structure is prepared to be acted as an electromagnetic attenuator. For instance, the string “5/0.5-4-5/0.5” in Fig. 5a refers to a three-layered composite with 5 mg Ti_3_C_2_ + 0.5 mg CNTs as the first/third layer and 4 mg CNFs as the second layer. Compared to the CNFs–CM–CNFs composite paper with same composition but different permutations of the layered structure, the CM–CNFs–CM paper with a wave-transmission intermediate layer exhibits an enhanced average EMI SE of 36 dB with a maximum of 37 dB. SE_Total_, SE_*R*_, and SE_*A*_ are calculated from the obtained scattering parameters to analyze the reason for EMI SE enhancement of CM–CNFs–CM paper. The increment in SE_*A*_ of CM–CNFs–CM composite paper relative to CNFs–CM–CNFs composite paper is greater than the increment in SE_*R*_, indicating that the higher effective absorption is responsible to the enhanced EMI shielding performance of CM–CNFs–CM paper (Fig. 5b). The content of CNFs between two CM layers in the CM–CNFs–CM composite paper is varied, and its effect on the EMI shielding performance is evaluated (Fig. 5c). With the CNFs content increasing from 2 to 4 mg, the average EMI SE exhibits a slight improvement from 35 to 36 dB, respectively. When the CNFs content further increased to 6 and 8 mg, the average EMI SE revealed a considerable reduction. It is mainly due to the existence of a small quantity of CNFs caused by vacuum-assisted filtration, which are involved in CM layer, and thus, the EMI performance is attenuated.

To investigate the relationship between gradient structures and the EMI SE of our composite paper, two-layered CM composite paper with various Ti_3_C_2_ contents in each layer has been fabricated with a sequential vacuum-assisted filtration method. Each composite paper is denoted with a string of numbers to conveniently represent the samples with different Ti_3_C_2_ contents. For example, the string “U10/1-S5/1” referred to two-layered composite with 10 mg Ti_3_C_2_ + 1 mg CNTs as the upper (first) layer and 5 mg Ti_3_C_2_ + 1 mg CNTs as the sublayer (second) layer. For the purpose of comparison, an evenly distributed “15/2” composite paper with 15 mg Ti_3_C_2_ + 2 mg CNTs has been fabricated via a same vacuum-assisted filtration method. As shown in Fig. 5d, although different gradient structures are presented in the samples, all these two-layered CM composite papers exhibit the similar EMI SE as SE_U10/1-S5/1_ ≈ SE_U5/1-S10/1_ ≈ SE_15/2_ over the whole X-band. To analyze the EMI shielding performance more intuitively, the reflection (*R*) and absorption (*A*) coefficients of different samples have also been investigated. As shown in Fig. 5e, f, the *R* and *A* of two-layered CM composite paper with two kinds of gradient structures are quite different. Moreover, the obtained *R* and *A* have a correlation of *A*_U5/1-S10/1_ > *A*_U10/1-S5/1_ and *R*_U5/1-S10/1_ < *R*_U10/1-S5/1_, which are corresponding to the representation of Fig. S16 (SE_*A* U5/1-S10/1_ > SE_*A* U10/1-S5/1_; SE_*R* U5/1-S10/1_ < SE_*R* U10/1-S5/1_) [38, 39, 41, 69–71]. These results demonstrate that the gradient structures do not show distinct effect on the total EMI shielding effectiveness but significantly affect the value of SE_*A*_ and SE_*R*_.

**Fig. 6:**
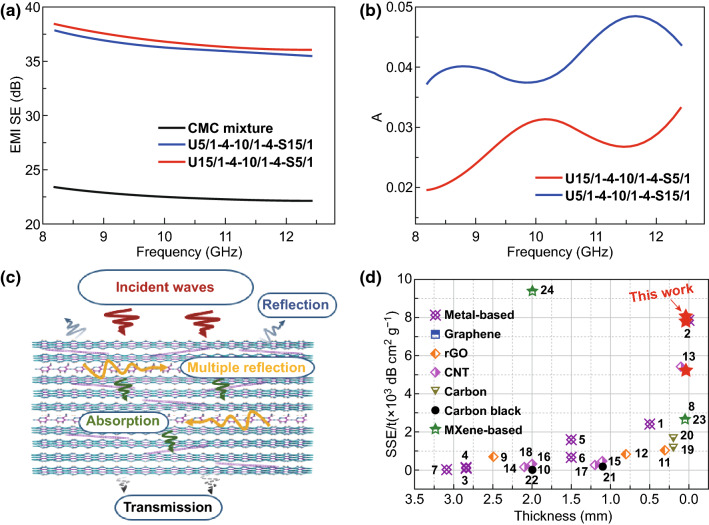
**a** EMI SE of randomly mixed CMC paper and CMC GS composite paper in the X-band. **b** The A of CMC GS composite paper with different gradient structures. **c** Proposed EMI shielding mechanism of the CMC GS composite paper. **d** Comparison of the specific EMI shielding effectiveness as a function of thickness. The numbers inside **d** are sample numbers listed in Table S2

To further investigate the influence of gradient and sandwich structures on the EMI shielding performance of multilayered CMC GS composite paper, two layers of CNFs and three layers of CM with various Ti_3_C_2_ contents have been used to be assembled into a composite paper for the analysis of EMI SE. Similarly, each composite paper has a specific string to conveniently represent the samples. For instance, the string “U5/1-4-10/1-4-S15/1” refers to a five-layered composite with CM-5 (5 mg Ti_3_C_2_ + 1 mg CNTs) as the first layer, 4 mg CNFs as the second/fourth layer, CM-10 (10 mg Ti_3_C_2_ + 1 mg CNTs) and CM-5 (15 mg Ti_3_C_2_ + 1 mg CNTs) as the third and fifth layer, respectively. As shown in Fig. 6a, the CMC GS composite paper with an average EMI SE about 36.6 dB exhibits a better EMI shielding performance than the randomly mixed CNTs/Ti_3_C_2_ MXene/CNFs composite paper (CMC mixture). Moreover, the five-layered CMC GS composite paper with opposite gradient structure shows nearly the same EMI SE as SE_U5/1-4-10/1-4-S15/1_ ≈ SE_U15/1-4-10/1-4-S5/1_ over the whole X-band. The highest EMI SE value of the CMC GS composite papers is 38.4 dB, indicating a high capability to block 99.99% of incident waves and only 0.01% transmission. Besides, the CMC GS composite paper with various gradient structures exhibits very different absorption coefficients, SE_*R*_, and SE_*A*_, which are similar to the results of the above-mentioned CM composite paper with gradient structures (Figs. 6b and S17). Additionally, the electrical conductivity of CMC GS composite paper and randomly mixed CMC composite paper has been also investigated. It can be seen from Fig. S18 that the conductivity of CMC GS composite paper with 2506.6 S m^−1^ is about 5 times higher than CMC mixture paper with 546.6 S m^−1^. All these results revealed a similar trend with the above-mentioned two- or three-layered composite paper, indicating that the composite paper with outstanding sandwich and gradient structure has a more adjustable EMI SE and microwave-absorbing property than the randomly mixed composite paper with uniform structure. A potential mechanism of the CMC GS composite paper for EMI shielding is proposed as illustrated in Fig. 6c. As the electromagnetic waves struck the surface of CMC GS composite paper, the reflection, absorption, and transmission of electromagnetic waves can occur. Firstly, some incident waves are immediately reflected at the interface between air and the CM layer owing to their impedance mismatch, which are mainly attributed to the existence of numerous free electrons at the surface of Ti_3_C_2_ MXene nanosheets and CNTs [72–74]. The oriented alignment of 2D Ti_3_C_2_ MXene nanosheets and 1D CNTs along the planar direction can achieve a continuous conductive path and endow the CM layer with high conductivity [75]. The most remaining waves interact with the high electron density of Ti_3_C_2_ MXene and CNTs, giving rise to ohmic losses and attenuating the energy of waves [3, 10, 26]. Additionally, the overall laminated structure enables the CM layer to behave as a multilevel shield, which can make the waves be reflected and forth between the adjacent MXene nanosheets until completely absorbed. Meanwhile, the high conductive CM layer with undulating layered structure and slit-shaped micropores, which are formed by using CNTs as spacers, will generate multiple internal reflections to promote the dissipation of electromagnetic waves. Polarization loss is also an important way to attenuate the incident waves. Actually, the localized defects of post-etched Ti_3_C_2_ MXene nanosheets can generate an asymmetry distribution of electrons and further lead to dielectric loss. Moreover, the terminating groups (–F, =O, or –OH) on the surface of MXene nanosheets can give rise to the asymmetric distribution of charge density, which promotes the formation of local dipoles. These dipoles will rotate directionally toward the electromagnetic field and result in polarization relaxation and electromagnetic energy loss in the form of heat, which in turn enhance the overall shielding effectiveness [39, 76]. Particularly, the proposed gradient and sandwich structure of the CMC composite paper can attenuate or eliminate the internal electromagnetic waves by repeated reflection and adsorption and further achieve an excellent EMI shielding performance.

The superiority of the ultrathin and flexible CMC GS composite paper over other shielding materials is emphasized by the comparison of their specific EMI shielding effectiveness (SSE), which incorporated three important parameters, that is, the EMI SE, density, and thickness. At present, metal- and carbon-based materials are the mainstream shielding materials. Although some promising progress in these materials has been made, few materials that simultaneously possess ultrathin thickness, flexibility, and excellent EMI shielding performance have been reported. As shown in Fig. 6d and Table S2, the ultrathin and flexible CMC GS composite paper shows both ultrathin thickness and high SSE and ranked the top at the comparison chart when comparing with other shielding materials, such as metal-based materials [3, 77–80], graphene [63], reduced graphene oxide [8, 81, 82], carbon nanotube [10, 83], and other MXene-based materials [27, 55] [84].

## Conclusions

In summary, we have fabricated an ultrathin and flexible CNTs/Ti_3_C_2_ MXene/CNFs composite paper with gradient and sandwich structure by a facile vacuum-assisted filtration method. The obtained CMC GS composite paper exhibits excellent mechanical properties and an outstanding combination of high tensile strength (97.9 MPa) and toughness (2.1 MJ m^−3^). The results indicate that the gradient structures can make a great difference to the contributions from reflection and absorption of the composite paper, rather than their total EMI SE. Meanwhile, the sandwich structures with a different thickness enhance the EMI SE of composite paper and promote the composite paper to block more electromagnetic waves energy. The CMC composite paper with gradient and sandwich structure displays a better EMI SE of 38.4 dB than the randomly mixed CMC composite paper of 23.4 dB. Thus, the ultrathin, flexibility CMC GS composite paper with outstanding EMI shielding performance will greatly widen the practical applications in the fields of wearable or portable electronic devices.

## Electronic supplementary material

Below is the link to the electronic supplementary material.

Supplementary material 1 (PDF 887 kb)

Supplementary material 2 (AVI 19758 kb)
