# Supplementary material for: Ultrathin and Flexible CNTs/MXene/Cellulose Nanofibrils Composite Paper for Electromagnetic Interference Shielding
Source: Nanomicro Lett. 2019 Sep 7;11:72. doi: 10.1007/s40820-019-0304-y (PMC7770921; doi:10.1007/s40820-019-0304-y)
Supplement: Supplementary file 1 — Supplementary material 1 (PDF 887 kb) [file 40820_2019_304_MOESM1_ESM.pdf]

Supporting Information for

Ultrathin and Flexible CNTs/MXene/Cellulose Nanofibrils Composite Paper for Electromagnetic Interference Shielding

Wentao Cao[1,2], Chang Ma[2], Shuo Tan[1], Mingguo Ma[2,] *, Pengbo Wan[3,] *, Feng Chen[1,] *

[1]Department of Orthopedics, Shanghai Tenth People's Hospital, Tongji University School of Medicine, Shanghai 200072, People's Republic of China

[2]Engineering Research Center of Forestry Biomass Materials and Bioenergy, Beijing Key Laboratory of Lignocellulosic Chemistry, College of Materials Science and Technology, Beijing Forestry University, Beijing 100083, People's Republic of China

[3]Center of Advanced Elastomer Materials, State Key Laboratory of Organic-Inorganic Composites, Beijing University of Chemical Technology, Beijing 100029, People's Republic of China

*Corresponding authors. E-mail: mg_ma@bjfu.edu.cn (Mingguo Ma); pbwan@mail.buct.edu.cn (Pengbo Wan); fchen@tongji.edu.cn (Feng Chen)

Supplementary Figures and Tables

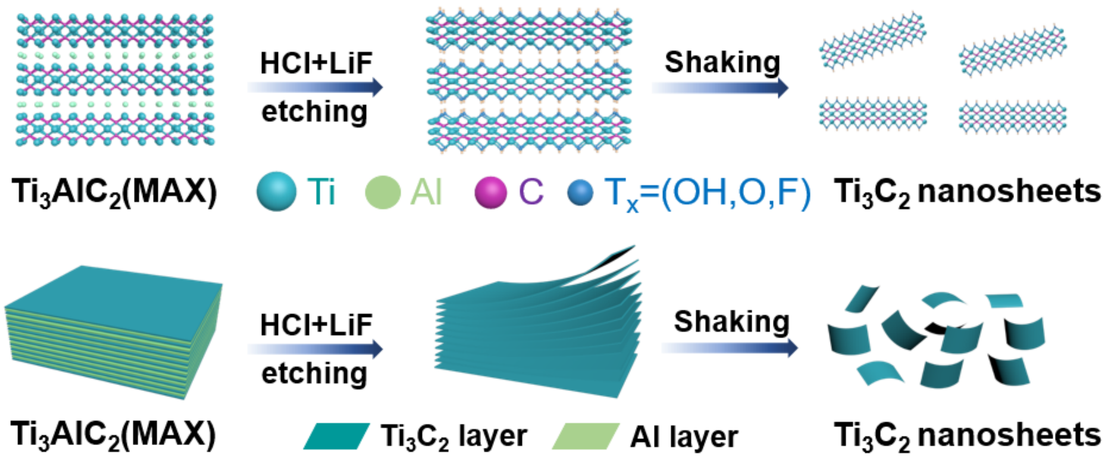

Fig. S1 Schematic illustration of the preparation of Ti_3C_2 nanosheets

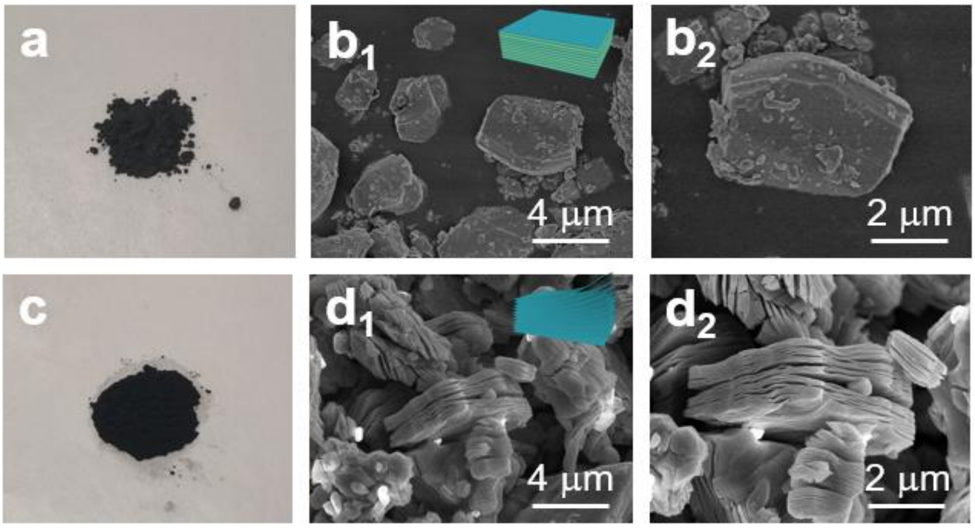

Fig. S2 a Digital image and **b** SEM images of Ti_3AlC_2 precursor (MAX phase). **c** Digital image and **d** SEM images of multilayered Ti_3C_2 MXene

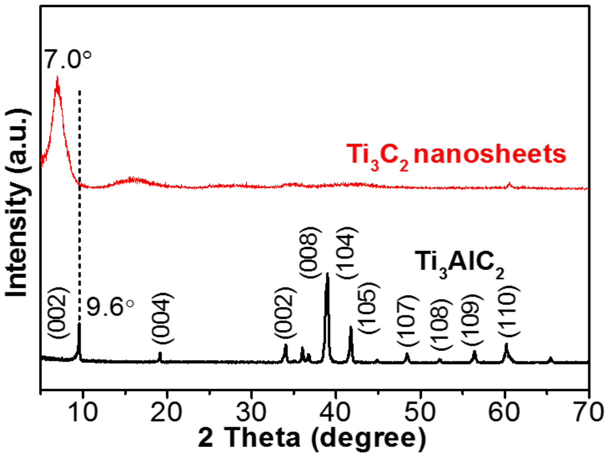

Fig. S3 XRD patterns of Ti_3AlC_2 precursor and Ti_3C_2 nanosheets

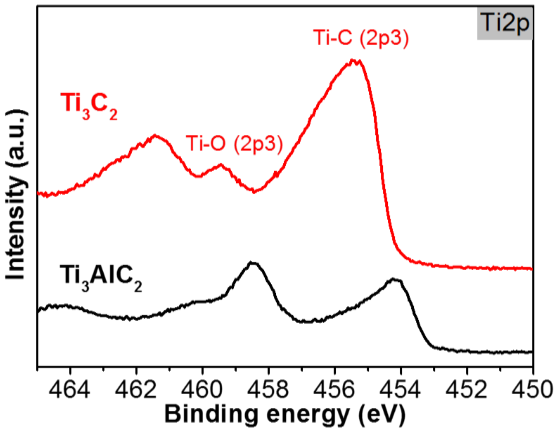

Fig. S4 Ti 2p spectra of Ti_3AlC_2 precursor and Ti_3C_2 nanosheets

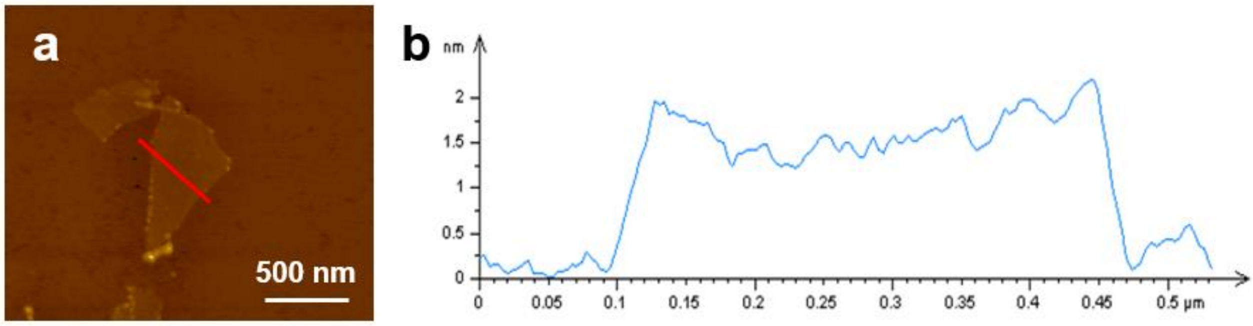

Fig. S5 a AFM image, and **b** height cutaway view of Ti_3C_2 nanosheets

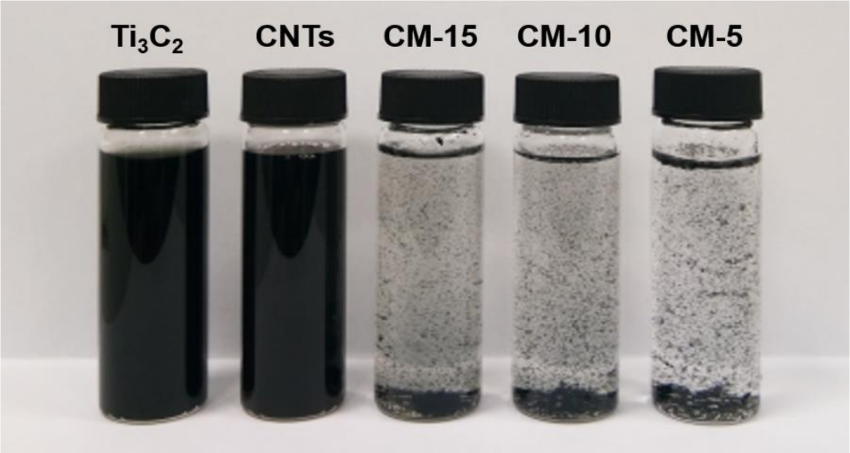

Fig. S6 Digital photographs of Ti_3C_2 suspension, CNTs aqueous dispersion, and CM composites with various Ti_3C_2 content

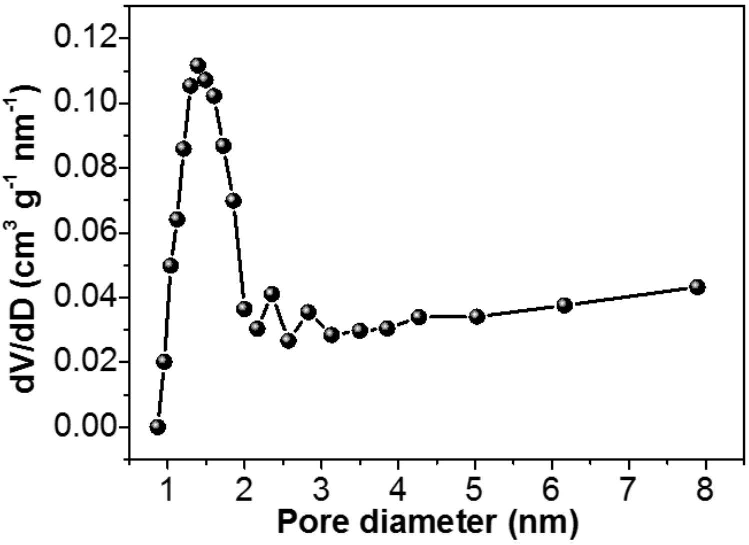

Fig. S7 The pore size distribution curve of CNTs

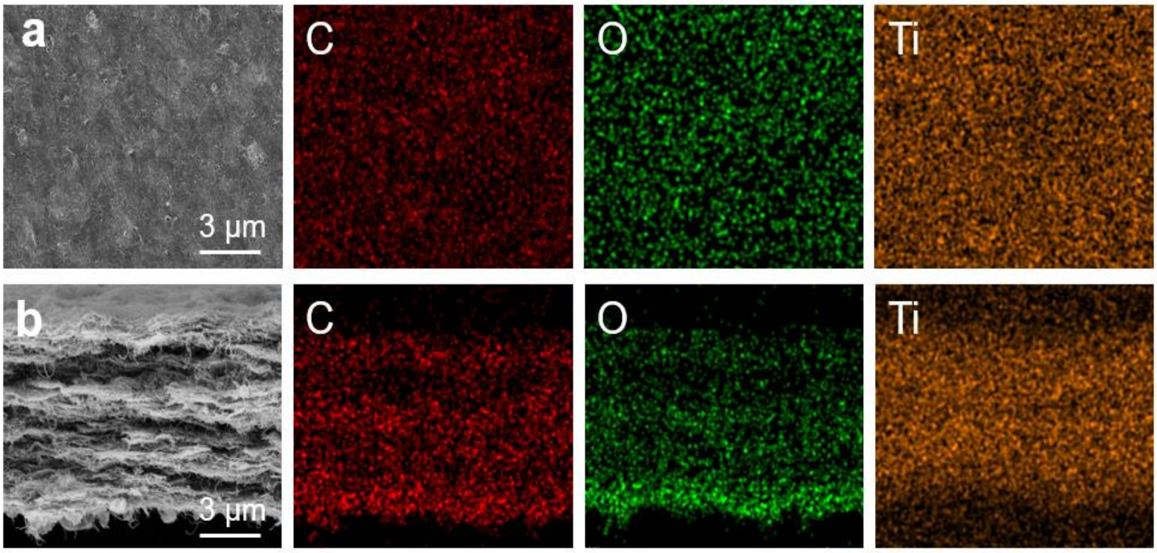

Fig. S8 a Top-view SEM and EDS mapping images of Ti_3C_2-CNTs composite paper. **b** Cross-sectional SEM and EDS mapping images of Ti_3C_2-CNTs composite paper

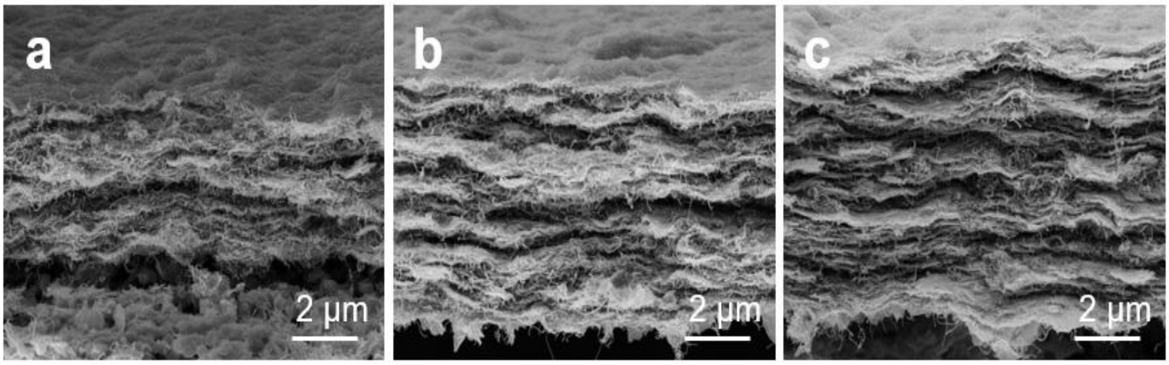

Fig. S9 Cross-sectional SEM images of **a** CM-5, **b** CM-10, and **c** CM-15

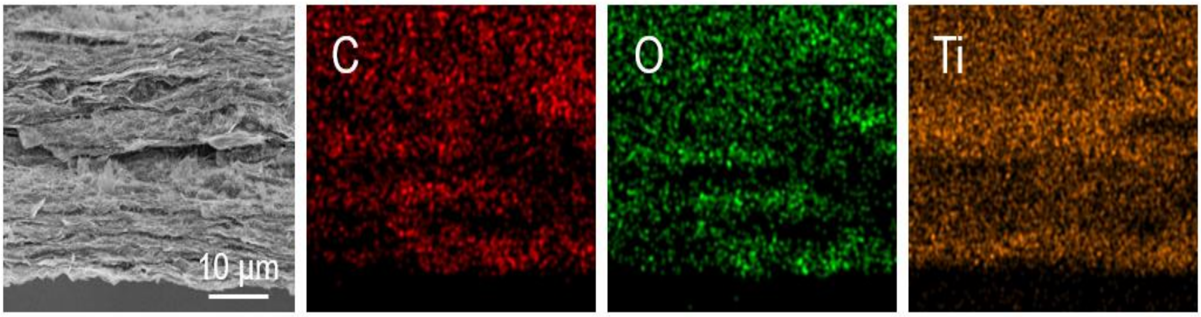

Fig. S10 Cross-sectional SEM and EDS mapping images of the free-standing CMC mixture composite paper

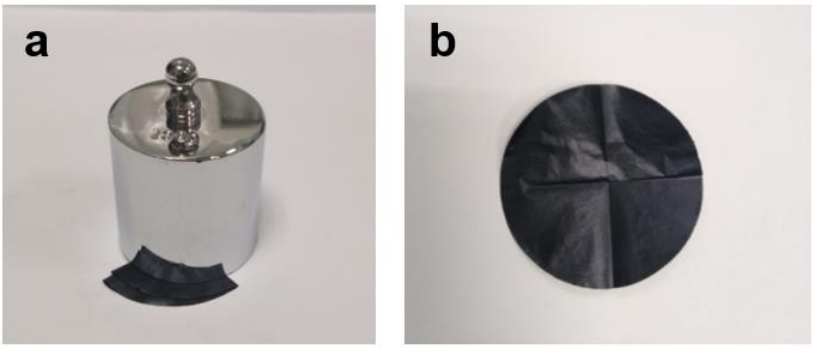

Fig. S11 a CMC GS composite paper was pressed with a weight of 200 g. **b** The CMC GS composite paper was not broken or cracked after pressed

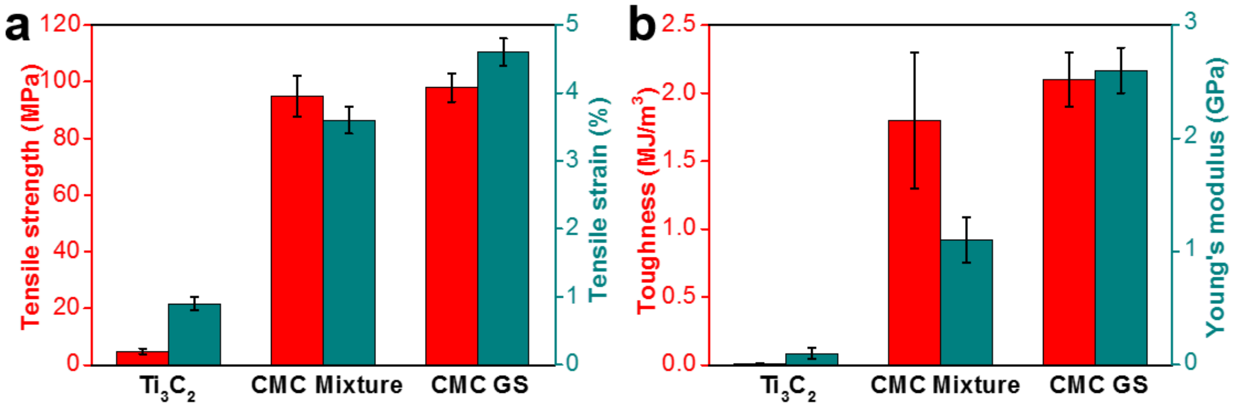

Fig. S12 a The tensile strengths and tensile strains of pure Ti_3C_2, CMC mixture, and CMC composite paper. **b** The toughness and young's modulus of pure Ti_3C_2, CMC mixture, and CMC composite paper

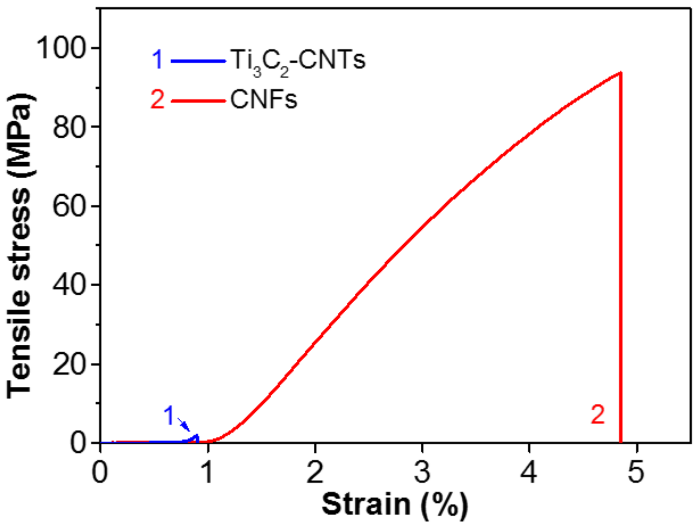

Fig. S13 Tensile stress-strain curves of the Ti_3C_2-CNTs composite paper and the pure CNFs paper

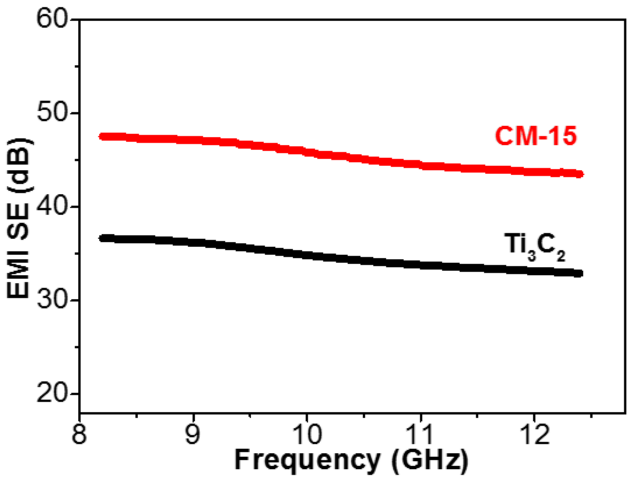

Fig. S14 The EMI SE of pure Ti_3C_2 paper and single-layered CM in the X-band region

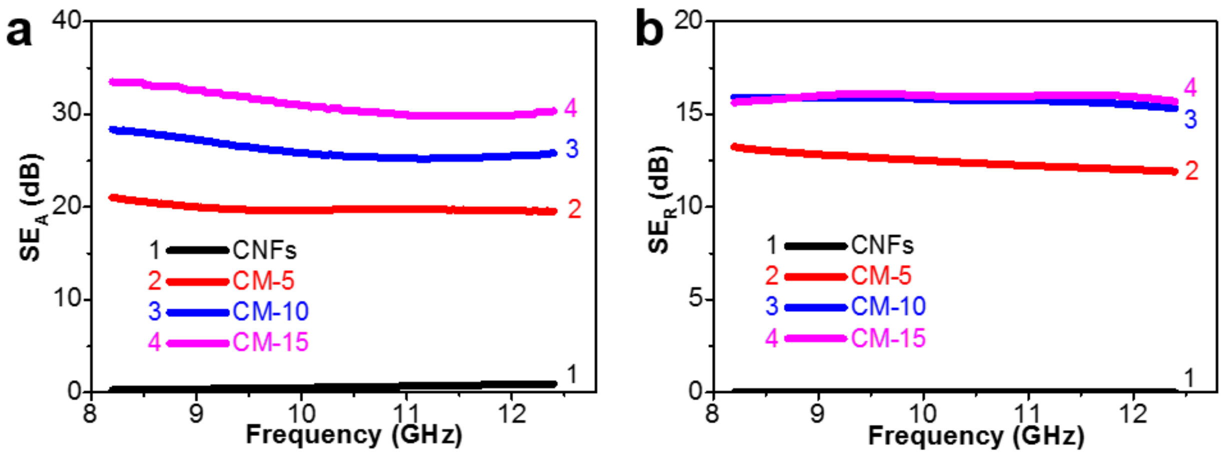

Fig. S15 a SE_A and **b** SE_R of single-layered TONFC, CM-5, CM-10, and CM-15 in the X-band region

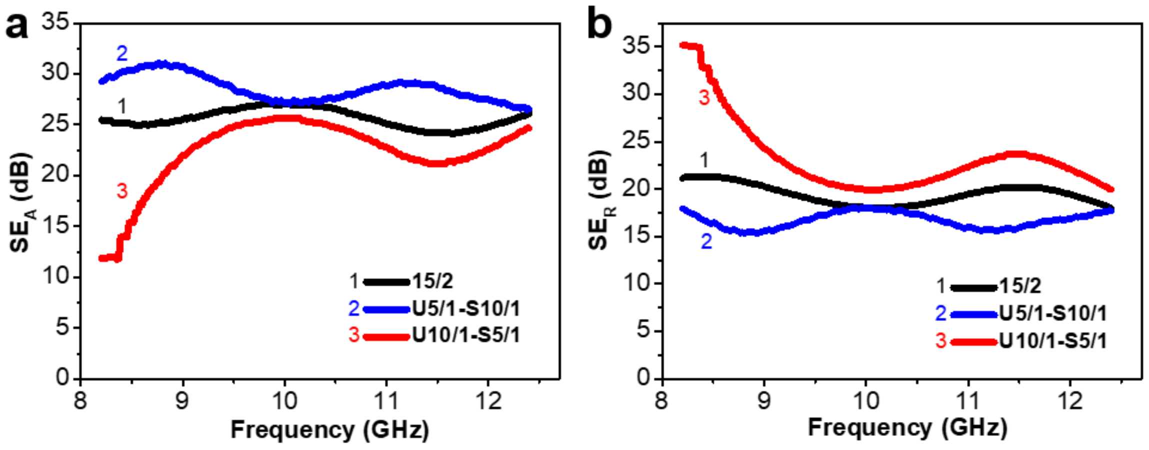

Fig. S16 a SE_A, and **b** SE_R of evenly distributed CM composite paper and two-layered CM composite paper with different gradient structures in the X-band

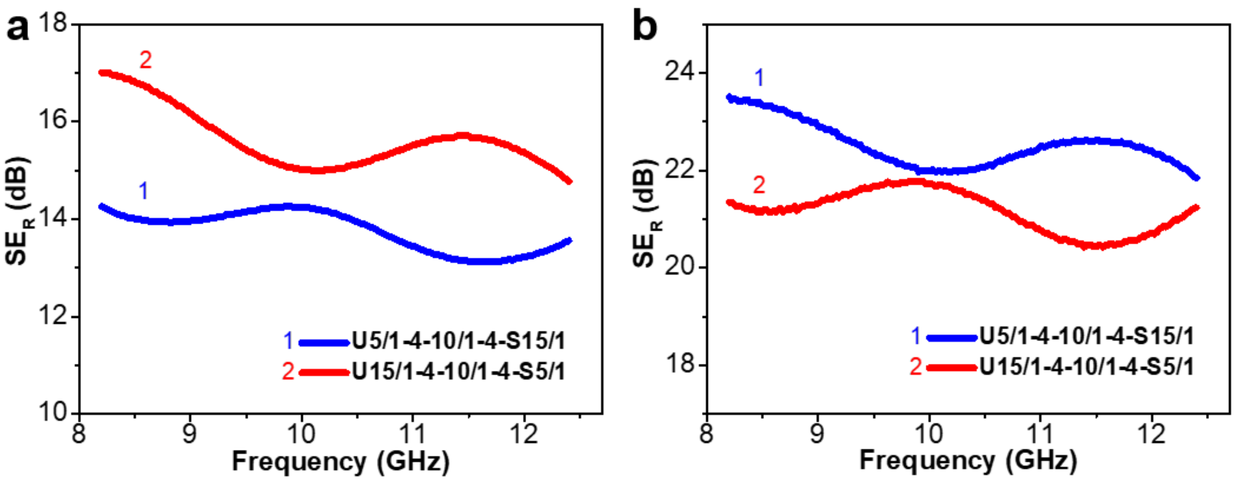

Fig. S17 a SE_R, and **b** SE_A of CMC GS composite paper with different gradient structures

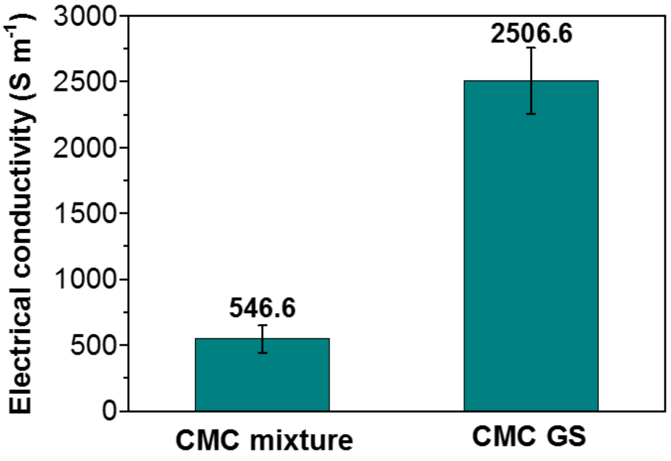

Fig. S18 The electrical conductivity of CMC mixture paper and CMC GS composite paper.

Table S1 The mechanical properties of different samples

Sample	Tensile strength (MPa)	Fracture strain (%)	Toughness (MJ m^{-3})	Young's modulus (GPa)
Ti_3C_2 MXene	4.9 ± 1.0	0.9 ± 0.1	0.010 ± 0.005	0.10 ± 0.05
Ti_3C_2-CNTs	2.4 ± 1.3	0.8 ± 0.4	0.0019 ± 0.0004	0.25 ± 0.1
CNFs	95.7 ± 13.7	5.1 ± 1.7	1.8 ± 0.5	2.5 ± 0.1
CMC mixture	94.9 ± 7.4	3.6 ± 0.2	1.8 ± 0.5	1.1 ± 0.2
CMC GS	97.9 ± 5.0	4.6 ± 0.2	2.1 ± 0.2	2.6 ± 0.2

Table S2 Comparison of the EMI shielding performance of the CMC GS composite paper with other reported materials

Type	Sample	Materials	Thickness (mm)	SE (dB)	SSE/t (dB cm^2 g^{-1})	Refs.
**Metal-based**	1	Ag NW	0.5	35	2416	[S1]
	2	Cu foil	0.0001	70	7812	[S2]
	3	Ni fiber/PES	2.85	58	109	[S3]
	4	Ni filaments/PES	2.85	87	165	[S4]
	5	CuNi-CNT	1.5	54.6	1580	[S5]
	6	CuNi foam	1.5	25	690	[S5]
	7	Copper	3.1	90	32	[S3]
**Carbon-based**	8	Graphene/PDMS	0.1	20	3330	[S6]
	9	rGO	2.5	45.1	692	[S7]
	10	rGO/PS	2	29	258	[S8]
	11	rGO/Fe_3O_4	0.3	24	1033	[S4]
	12	rGO/PEDOT	0.8	70	841	[S9]
	13	MWCNT/WPU	0.1	21.1	5410	[S10]
	14	MWCNT/PC	2.1	39	164	[S11]
	15	MWCNT/ABS	1.1	50	433	[S12]
	16	MWCNT/PS	2	30	285	[S13]
	17	SWCNT/PS	1.2	18.5	275	[S14]
	18	SWCNT/epoxy	2	25	72	[S15]
	19	Carbon foam	0.2	40	1250	[S16]
	20	Carbon/PN resin	0.2	51.2	1705	[S17]
	21	CB/ABS	1.1	20	190	[S12]
	22	CB/EPDM	2	18	15	[S18]
**MXene-based**	23	$Ti_3C_2T_x$/CNFs	0.047	24	2647	[S19]
	24	$Ti_3C_2T_x$/rGO/epoxy	2	56.4	9400	[S20]
**This work**	25	CMC mixture	0.038	23.4	5219	
	26	CMC GS-1	0.038	37.7	7874	
	27	CMC GS-2	0.038	38.4	8020	

NW: nanowire; PES: polyethersulfone; CNT: carbon nanotube; PDMS: poly(dimethyl siloxane); rGO: reduced grapheme oxide; MWCNT: multi-walled carbon nanotube; SWCNT: single-walled carbon nanotube; PS: polystyrene; WPU: water-borne polyurethane; CNFs: cellulose nanofibers; CMC GS-1: U5/1-4-10/1-4-S15/1; CMC GS-2: U15/1-4-10/1-4-S5/1

Supplementary References

[S1] J. Ma, K. Wang, M. Zhan, A comparative study of structure and electromagnetic interference shielding performance for silver nanostructure hybrid polyimide foams. RSC Adv. **5**(80), 65283-65296 (2015). https://doi.org/10.1039/c5ra09507g

[S2] F. Shahzad, M. Alhabeb, C.B. Hatter, B. Anasori, S.M. Hong, C.M. Koo, Y. Gogotsi,

Electromagnetic interference shielding with 2D transition metal carbides (MXenes). Science **353**(6304), 1137-1140 (2016). https://doi.org/10.1126/science.aag2421

[S3] X.P. Shui, D.D.L. Chung, Nickel filament polymer-matrix composites with low surface impedance and high electromagnetic interference shielding effectiveness. J. Electron. Mater. **26**(8), 928-934 (1997). https://doi.org/10.1007/s11664-997-0276-4

[S4] W.-L. Song, X.-T. Guan, L.-Z. Fan, W.-Q. Cao, C.-Y. Wang, Q.-L. Zhao, M.-S. Cao, Magnetic and conductive graphene papers toward thin layers of effective electromagnetic shielding. J. Mater. Chem. A **3**(5), 2097-2107 (2015). https://doi.org/10.1039/c4ta05939e

[S5] K. Ji, H. Zhao, J. Zhang, J. Chen, Z. Dai, Fabrication and electromagnetic interference shielding performance of open-cell foam of a cu-ni alloy integrated with CNTs. Appl. Surf. Sci. **311**(351-356 (2014). https://doi.org/10.1016/j.apsusc.2014.05.067

[S6] Z. Chen, C. Xu, C. Ma, W. Ren, H.-M. Cheng, Lightweight and flexible graphene foam composites for high-performance electromagnetic interference shielding. Adv. Mater. **25**(9), 1296-1300 (2013). https://doi.org/10.1002/adma.201204196

[S7] D.-X. Yan, H. Pang, B. Li, R. Vajtai, L. Xu, P.-G. Ren, J.-H. Wang, Z.-M. Li, Structured reduced graphene oxide/polymer composites for ultra-efficient electromagnetic interference shielding. Adv. Funct. Mater. **25**(4), 559-566 (2015). https://doi.org/10.1002/adfm.201403809

[S8] D.-X. Yan, P.-G. Ren, H. Pang, Q. Fu, M.-B. Yang, Z.-M. Li, Efficient electromagnetic interference shielding of lightweight graphene/polystyrene composite. J. Mater. Chem. **22**(36), 18772-18774 (2012). https://doi.org/10.1039/c2jm32692b

[S9] N. Agnihotri, K. Chakrabarti, A. De, Highly efficient electromagnetic interference shielding using graphite nanoplatelet/poly(3,4-ethylenedioxythiophene)-poly(styrenesulfonate) composites with enhanced thermal conductivity. RSC Adv. **5**(54), 43765-43771 (2015). https://doi.org/10.1039/c4ra15674a

[S10] Z. Zeng, H. Jin, M. Chen, W. Li, L. Zhou, Z. Zhang, Lightweight and anisotropic porous MWCNT/WPU composites for ultrahigh performance electromagnetic interference shielding. Adv. Funct. Mater. **26**(2), 303-310 (2016). https://doi.org/10.1002/adfm.201503579

[S11] S. Pande, A. Chaudhary, D. Patel, B.P. Singh, R.B. Mathur, Mechanical and electrical properties of multiwall carbon nanotube/polycarbonate composites for electrostatic discharge and electromagnetic interference shielding applications. RSC Adv. **4**(27), 13839-13849 (2014). https://doi.org/10.1039/c3ra47387b

[S12] M.H. Al-Saleh, W.H. Saadeh, U. Sundararaj, EMI shielding effectiveness of carbon based nanostructured polymeric materials: A comparative study. Carbon **60**, 146-156 (2013).

https://doi.org/10.1016/j.carbon.2013.04.008

[S13] M. Arjmand, T. Apperley, M. Okoniewski, U. Sundararaj, Comparative study of electromagnetic interference shielding properties of injection molded versus compression molded multi-walled carbon nanotube/polystyrene composites. Carbon **50**(14), 5126-5134 (2012). https://doi.org/10.1016/j.carbon.2012.06.053

[S14] Y.L. Yang, M.C. Gupta, Novel carbon nanotube-polystyrene foam composites for electromagnetic interference shielding. Nano Lett. **5**(11), 2131-2134 (2005). https://doi.org/10.1021/nl051375r

[S15] Y. Huang, N. Li, Y. Ma, D. Feng, F. Li et al., The influence of single-walled carbon nanotube structure on the electromagnetic interference shielding efficiency of its epoxy composites. Carbon **45**(8), 1614-1621 (2007). https://doi.org/10.1016/j.carbon.2007.04.016

[S16] F. Moglie, D. Micheli, S. Laurenzi, M. Marchetti, V.M. Primiani, Electromagnetic shielding performance of carbon foams. Carbon **50**(5), 1972-1980 (2012). https://doi.org/10.1016/j.carbon.2011.12.053

[S17] L. Zhang, M. Liu, S. Roy, E.K. Chu, K.Y. See, X. Hu, Phthalonitrile-based carbon foam with high specific mechanical strength and superior electromagnetic interference shielding performance. ACS Appl. Mater. Interfaces **8**(11), 7422-7430 (2016). https://doi.org/10.1021/acsami.5b12072

[S18] P. Ghosh, A. Chakrabarti, Conducting carbon black filled edpm vulcanizates: Assessment of dependence of physical and mechanical properties and conducting character on variation of filler loading. Eur. Polym. J. **36**(5), 1043-1054 (2000). https://doi.org/10.1016/s0014-3057(99)00157-3

[S19] W.-T. Cao, F.-F. Chen, Y.-J. Zhu, Y.-G. Zhang, Y.-Y. Jiang, M.-G. Ma, F. Chen, Binary strengthening and toughening of MXene/cellulose nanofiber composite paper with nacre-inspired structure and superior electromagnetic interference shielding properties. ACS Nano **12**(5), 4583-4593 (2018). https://doi.org/10.1021/acsnano.8b00997

[S20] S. Zhao, H.-B. Zhang, J.-Q. Luo, Q.-W. Wang, B. Xu, S. Hong, Z.-Z. Yu, Highly electrically conductive three-dimensional $Ti_3C_2T_x$ mxene/reduced graphene oxide hybrid aerogels with excellent electromagnetic interference shielding performances. ACS Nano **12**(11), 11193-11202 (2018). https://doi.org/10.1021/acsnano.8b05739
